# Effect of Binaural Beats on Affective Symptoms and Performance on the Digit Span Test

**DOI:** 10.7759/cureus.94182

**Published:** 2025-10-09

**Authors:** Blair Chen, Christine Wong, Regina Dzebley, James M Stone

**Affiliations:** 1 Clinical Neuroscience, Brighton and Sussex Medical School, Brighton, GBR

**Keywords:** anxiety, binaural beats, dass-21, depression, working memory

## Abstract

Background

Binaural beats (BB) are auditory tones perceived in the brain when two sine waves with different frequencies are played into each ear. Previous studies have suggested potential benefits of using BB for improving concentration and reducing anxiety and stress. In this study, we investigated the effect of theta (6 Hz), alpha (10 Hz), and beta (16 Hz) BB on measures of stress, anxiety, and short-term and working memory.

Methods

Sixty-three healthy participants were recruited. They were randomly allocated to a different sub-study in which they listened to (1) 6 Hz (n=14), (2) 10 Hz (n=14), or (3) 16 Hz (n=14) BB centred around 340 Hz, or a 340 Hz control tone (n=21), with the pink noise set at 60% and the tones at 40%. Before and after the audio, participants had their blood pressure and heart rate measured, completed a digit span task, and completed the Depression, Anxiety, and Stress Scale-21 items (DASS-21). Electrodermal activity (EDA) data was collected before and during the audio. We compared the percentage change between baseline and during audio exposure for all measures between the BB groups and control group using the Wilcoxon signed-rank test.

Results

There were no significant effects of any BB on blood pressure, heart rate, or skin conductance nor on performance on the digit span test. DASS-21 total scale showed a trend for reduction in alpha frequency only (p<0.1). Post hoc analyses of subscales revealed a reduction in stress with alpha frequency (p=0.022) and a trend for reduction in depression (p=0.09). Theta showed a trend for an increase in stress (p=0.06).

Conclusions

Further work is required to determine whether the effects of alpha BB on stress ratings are consistently found. It is possible that BB may be useful adjuncts to clinical treatments in the future.

## Introduction

Binaural beats (BB) are a phenomenon in which a third pulsing tone is perceived in the brain by the simultaneous presentation of two sinusoidal waves with slightly different frequencies to each ear. The beating tone produced in this way has a frequency of the difference of the two frequencies presented [1], by a process known as binaural integration [2]. For instance, if a 340 Hz tone is presented to the left ear and a 350 Hz tone is presented to the right ear simultaneously, the BB generated will be 10 Hz. Generally, the two tones are required to be less than 1000 Hz with smaller than 30 Hz difference for BB to be produced [3].

BB may induce brainwave entrainment, a phenomenon in which brain waves synchronise with an external stimulus, leading to a frequency-following response (FFR) in the brain, where the frequency of brain activity matches the frequency of the stimulus [4]. FFR is an auditory evoked response that can be recorded with an electroencephalogram (EEG) and provides information on sound processing [5]. Several studies report that the BB-related brainwave entrainment can alter neuronal responses and can therefore change the connectivity between different brain regions and cortical networks [6]. Other researchers have challenged the entrainment theory and suggested that the changes could be due to the neural oscillations of the two hemispheres being synchronised, in other words, an increase of interhemispheric coherence which has been found with alpha and theta BB [7].

Although the exact mechanism behind BB is still unclear, the psychophysiological effects of different BB frequencies have been explored by various research. For example, anxiolytic [6,8] and analgesic [9] effects were found in alpha and theta BB, respectively. Previous work has suggested that gamma BB were the most beneficial frequency for improving attention [10], although this has not been consistently replicated [11]. Another group reported that alpha and gamma frequency BB increased creativity in participants with higher dopamine levels [12]. Many other studies did not find evidence for any mood modulation or psychophysiological change after BB intervention [13-15].

Despite the conflicting findings, it has been suggested that BB may be a useful tool to alter cognitive function and emotional states. A variety of benefits such as enhancing attention, memory, and meditation as well as reducing anxiety, stress, and pain have been claimed. Therefore, BB are also utilised as an alternative therapy for anxiety or as digital drugs to mimic the effects of psychoactive drugs by their supporters [11,16]. A meta-analysis by Garcia-Argibay et al. and a recent systematic review by Basu and Banerjee both found encouraging but mixed results on the claimed benefits from previous trials indicating more research is warranted with more consistent experimental variables like exposure time, tool to assess cognitive function, and masking sound [6,10].

In this study, we aimed to investigate three different frequencies of BB, theta, alpha and beta, to determine whether they had any effect on short-term and working memory, as indexed with the digit span test, and also whether they had any effect on measures of stress and anxiety, as measured using the Depression, Anxiety, and Stress Scale-21 items (DASS-21), or on blood pressure or skin conductance.

## Materials and methods

Participants

Sixty-three healthy, adult participants were recruited by verbal invitation and advertisement on social media. All participants were aged 18 and above and had normal or corrected-to-normal vision. Exclusion criteria included individuals with a medical history of hearing impairment in one or both ears or a history of epilepsy. The term "auditory tones" was used throughout the study instead of the term "binaural beats" to maintain the integrity of the control group of the study. All participants were provided with a participant information sheet prior to the experiment, followed by consent forms which were signed by the participants. All participants were financially compensated with £10 for taking part in this study.

Ethics statement

The protocol and financial compensation for the study were reviewed and granted approval by the Brighton and Sussex Medical School Research Governance and Ethics Committee (RGEC) (approval number: ER/BSMS9H84/1). All procedures were carried out in accordance with the approved protocol, and ethical standards were upheld throughout the study.

Randomisation procedure

This study was a double-blinded randomised controlled trial. Participants were recruited into one of the three BB frequency groups (alpha, beta, or theta), with 21 participants recruited into each group. Within each group, participants were further allocated into one of the three groups. Two out of the three groups were the active groups, whereas the other was the control.

BB stimuli

The auditory stimuli involved using a combination of pink noise with varying auditory tones depending on the BB frequency groups and the control group. The alpha BB audio file consisted of a 345 Hz wave on the left side and a 335 Hz wave on the right side, generating a 10 Hz alpha BB. The beta BB audio file consisted of a 348 Hz wave on the left side and a 332 Hz wave on the right side, generating a 16 Hz beta BB. The theta BB audio file consisted of a 343 Hz wave on the left side and a 337 Hz wave on the right side, generating a 6 Hz theta BB. The control audio file consisted of a 340 Hz wave being projected into both ears. For all active BB audio files and the control audio file, the pink noise was set to a volume of 60%, and the auditory tones were set to a volume of 40%.

Digit span task

The forward digit span task is a widely used, validated tool to assess short-term and working memory. Participants are required to memorise a sequence of numbers shown on a computer screen and to type the sequence out immediately after. We used a JavaScript-designed, browser-based digit span task. In our task, the sequence of numbers increased in length each round until two consecutive mistakes were made by the participant, at which point the sequence length reduced by one. A subsequent mistake further reduced the sequence length by one. The digit span task used included a practice trial of three sequences to allow participants to familiarise themselves with the test and the device used. The practice sequences were followed by 14 test sequences starting at three digits. Forward recall only was tested. We scored the task as the maximum number of digits remembered in one go during the task.

Procedure

The study was conducted in-person in a quiet room. All participants were provided with a participant information sheet before completing a consent form. Participants were asked to rate their mood using the DASS-21 questionnaire. An initial reading of the participants' blood pressure and heart rate was recorded. Sensors of the "Shimmer" device (Shimmer Research Ltd., Dublin, Ireland) were then attached to the fingers of the participants to collect electrodermal activity (EDA) data. The sensors remained on the participants' fingers until the end of the experiment.

Following this, participants were then asked to carry out the digit span task on an electronic device such as a computer. The maximum length of the sequence of numbers recalled by each participant was recorded. Once the digit span test had been completed, participants were given a set of headphones which played the assigned audio file. Participants were exposed to the audio file for a duration of 15 minutes. During the first five minutes, participants were told to sit and relax while listening to the audio file. After five minutes, participants were asked to complete the digit span task again while listening to the audio file. Upon completion of the digit span task, participants were told to continue listening to the audio file for the rest of the time until the 15 minutes was completed. Participants then completed the DASS-21 questionnaire again, rating their mood after listening to the audio file. Finally, measurement of the blood pressure and heart rate of the participants was repeated.

Data collection and analysis

The time points at which the EDA data collection began, the start of the audio file, and the end of the audio file were marked against the EDA data recorded on the Consensys software (Shimmer Research Ltd., Dublin, Ireland) to identify the periods before and during the exposure to the audio files. We selected the conductance data reported as µS. This data was then processed in Ledalab to only include the relevant time periods and to remove artefacts. Change in tonic EDA (between baseline and BB exposure) for each BB frequency was calculated. DASS-21 subscale scores (depression, anxiety, and stress) were calculated.

Statistical analyses were carried out using R version 4.3.1 (R Foundation for Statistical Computing, Vienna, Austria). As the data were not normally distributed, we used the Wilcoxon signed-rank test to compare the differences in the data collected between the BB groups and the control group.

## Results

Sixty-three participants were recruited (47 female, 16 male) with a mean (SD) age of 24 (8.1). There was no significant difference in gender or age between groups (chi-square=7.20; df=3; p=0.07; F(3,59)=1.50; p=0.22) (Table 1).

**Table 1:** Participant demographics by the binaural beats group.

	Theta	Alpha	Beta	Control	
Gender (f/m)	10/4	8/6	14/0	15/6	Chi-square=7.20; df=3; p=0.07
Age (years) mean (SD)	26.9 (11.9)	22 (1.8)	22 (1.1)	24 (9.6)	F(3,59)=1.50; p=0.22

**Figure 1:**
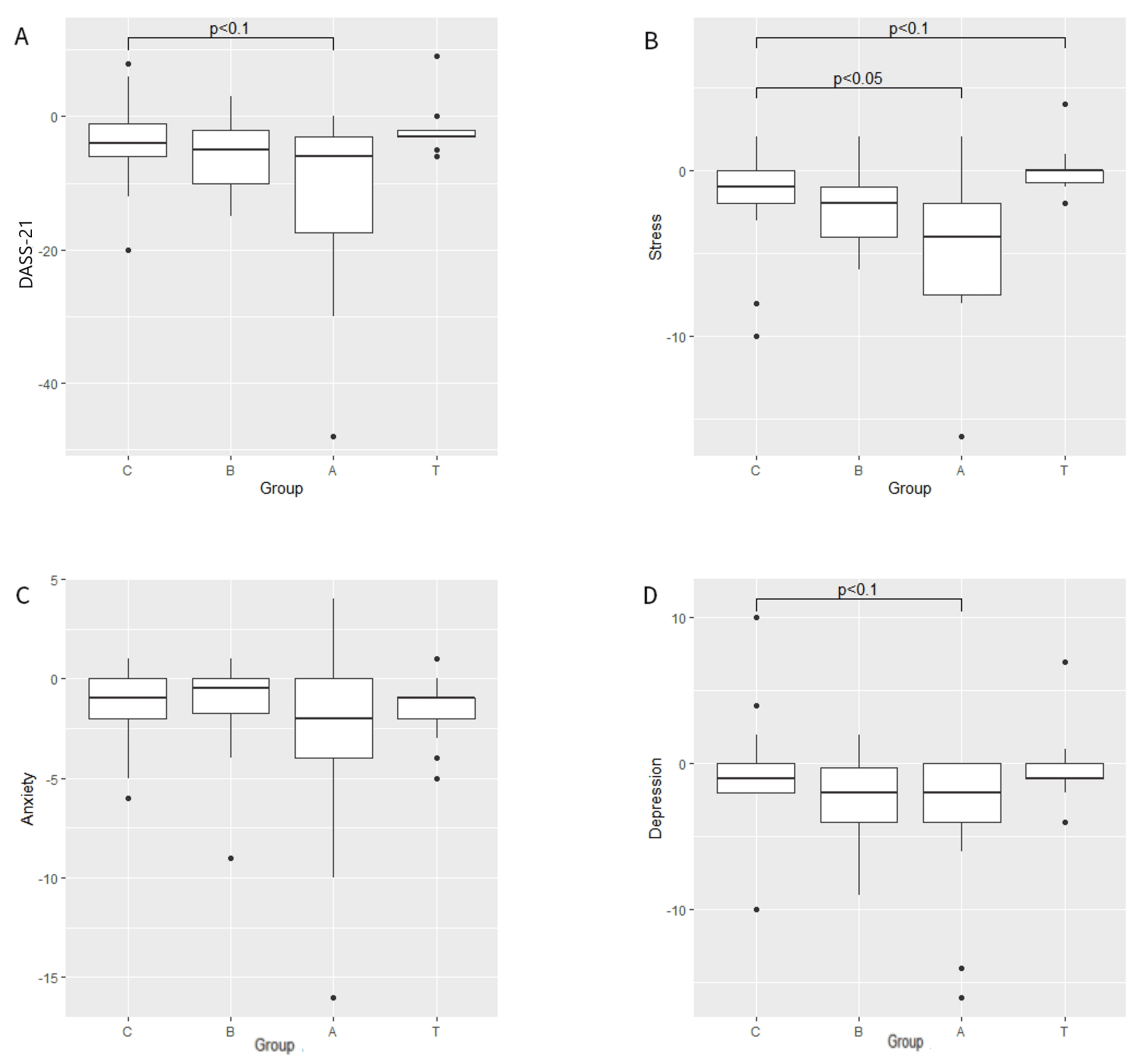
Change in DASS-21 (a), stress (b), anxiety (c), and depression (d) subscales before and after control or binaural beats exposure. DASS-21: Depression, Anxiety, and Stress Scale-21 items; C: control; B: beta; A: alpha; T: theta

There were no significant effects of any BB on blood pressure, heart rate, or skin conductance (Table 2). There was also no effect of BB exposure on performance on the digit span test (Table 3). DASS-21 total scale showed a trend for reduction in alpha frequency only (W=190; p<0.1) (Figure 1). Post hoc analyses of subscales revealed a reduction in stress with alpha frequency (W=214; p=0.022) (Figure 1) and a trend for reduction in depression (W=196; p=0.09) (Figure 1). Theta showed a trend for an increase in stress (W=94; p=0.06) (Figure 1). There were no effects of BB on anxiety (Figure 1).

**Table 2:** Blood pressure, heart rate, and skin conductance by the binaural beats group. ns: not significant

	Pre	Post	Statistics (vs. control)
Blood pressure (mmHg)			
Theta	121/72	113/70	p=ns
Alpha	122/76	116/75	p=ns
Beta	113/75	108/73	p=ns
Control	119/76	112/72	
Heart rate (bpm)			
Theta	72.4	69.9	p=ns
Alpha	72.7	73.5	p=ns
Beta	82.7	79.6	p=ns
Control	91.9	86.0	
Skin conductance (µS)			
Theta	1.64	2.45	p=ns
Alpha	3.19	3.88	p=ns
Beta	2.26	2.61	p=ns
Control	2.7	3.45	

**Table 3:** Performance on the digit span test by the binaural beats group. ns: not significant

	Pre	Post	Statistics (vs. control)
Theta: median (range)	6.5 (5-8)	7 (6-9)	p=ns
Alpha: median (range)	6.5 (5-8)	7 (5-10)	p=ns
Beta: median (range)	7 (5-8)	7 (5-10)	p=ns
Control: median (range)	7 (5-9)	8 (5-9)	

## Discussion

The primary finding of this pilot study was that the alpha frequency BB led to a significant reduction in the stress subscale of DASS-21. There was no effect of BB on performance on the digit span task. None of the physiological measures demonstrated a significant difference between the active and control groups, although blood pressure and heart rate decreased in all groups. This may be due to participants acclimatising to the environment by the time the second measurement was taken. It may have been beneficial to allow participants to sit for a set time before doing these measurements at the start of the experiment. International recommendations suggest 3-5 minutes of resting time is required before blood pressure measurement [17]. However, other studies have found that BB did not lead to any reduction in heart rate or blood pressure compared to placebo [18,19]. On the other hand, a number of studies indicate that auditory stimuli in general do result in a reduction in these measures, and so the effect of reduction in heart rate and blood pressure in the control group as well as the BB groups was not unexpected [20-22].

It is interesting to note that there was an overall increase in the skin conductance results for each frequency including the control group. This was consistent with previously reported findings [13]. A rise in tonic skin conductance has been shown to follow the level of stress, be that cognitive or emotional [23]. In our study, all participants completed a digit span task, which may have led to an increase in cognitive stress.

We found an overall reduction in depression, anxiety, and stress in DASS-21 in all groups including control. This may be because listening to slow, non-distressing sound promotes relaxation compared to silence [24]. In contrast to other groups [8,25,26], we did not find an overall effect of BB on anxiety, but we did find a reduction in stress for the alpha frequency BB.

It has been suggested that exposure of BB prior to the task as well as prior and during the task generates better results than exposure during the task only [6]. In this study, participants first listened to five minutes of audio and then did the digit span task with the audio continued, implementing the recommended exposure timing for a superior outcome.

There are some strengths of this study in that it included three treatment groups with a double-blinded placebo-controlled design. Neither participants nor researchers were aware of which group they were allocated to, and BB were not explicitly mentioned in any of the study-related materials for participants. 

There are, however, several limitations. We included a relatively small sample size of 21 participants for each frequency, which leads to an increased risk of type 2 error. The study was designed as a pilot to screen several different frequencies of BB, but future studies would require larger sample sizes in order to have the power to detect smaller effect sizes. Also, related to this issue, we did not include a correction for multiple comparisons. If we had done so, none of the findings would have remained significant.

Another issue is that a high proportion of participants in the study were students of similar age and background. There was a preponderance of females in all groups, and in the beta group, there were no males. This is an important consideration as a previous study showed that females and males have different perceptions of BB [27].

None of the participants was reported to have a mental illness, and so we would not expect any of them to score particularly highly on the DASS-21 scale. Furthermore, DASS-21 is designed for use over a longer time scale and may be less sensitive to changes such as the current study, where the intervention was only a one-off, 15-minute session.

Lastly, the short exposure time of BB may have impacted the findings. The optimal exposure time for reducing anxiety and improving attention for BB stimulation has been suggested to be 20-30 minutes [28].

## Conclusions

BB provide a potentially non-invasive and cost-effective method of modulating emotion and concentration. It is possible that BB may be useful adjuncts to clinical treatments in the future. Future work from our group will further investigate the effect of BB in larger sample sizes and incorporate the combined frequency of BB. We will also investigate the potential utility of BB in other situations such as improving sleep and reducing stress responses.
